# Spatiotemporal hotspot analysis of tuberculosis lost to follow-up cases in Ghana: A district-level study from 2019–2023

**DOI:** 10.1371/journal.pone.0326444

**Published:** 2025-07-02

**Authors:** David Ngmenbelle, Baaba Amoa-Saah, Stephen Nimirkpen, Maxwell Adjiabadek Akesem, Jacob Solomon Idan, Aliyu Mohammed

**Affiliations:** 1 Department of Epidemiology and Biostatistics, School of Public Health, Kwame Nkrumah University of Science and Technology, Kumasi, Ghana; University of Dschang, CAMEROON

## Abstract

**Introduction:**

Despite existing measures to control Tuberculosis (TB), the burden of TB remains a serious challenge in Ghana, with declining treatment success rates over recent years. Lost to Follow-Up (LTFU) has been attributed to this decline. This cross-sectional study aims to employed spatiotemporal analysis, an underutilized approach in this context, to explore areas with high prevalence of LTFUs in order to improve TB treatment success rates.

**Methods:**

A spatiotemporal analysis was conducted in Ghana using TB LTFU data from 2019–2023 extracted from the District Health Information Management System 2 (DHIMS2). Proportions of LTFU were used for spatial mapping. We adopted the Global Moran’s I, LISA and Getis-Ord G* techniques to determine spatial autocorrelation, optimized clusters or outliers and identify hotspot areas respectively.

**Results:**

A total of 2,887 TB LTFU cases were recorded out of 75,604 TB cases. We observed an initial increase of TB LTFU from 2019 (4.12%) to 2020, and a diminishing trend (5.28% to 3.11%) from 2020 to 2023. The Global Moran’s I estimations showed significant spatial clustering of TB LTFU cases from 2019 to 2021, shifting to a more random distribution in 2022 and 2023. High spatial clustering of LTFU were primarily reported in districts within Eastern, Central, and Greater Accra regions across 2019–2023, with clusters in Volta and Ashanti regions in 2021. We identified significant hotspot areas in districts within Greater Accra, Central, and Eastern regions.

**Conclusions:**

Hotspot areas of TB LTFU were primarily identified in densely populated regions. Strategic plans such as intensive education programs should be implemented to address pertinent issues regarding LTFU in the affected districts. Priorities should be directed towards populated regions, particularly Greater Accra, Central and Eastern regions, to improve TB treatment adherence and outcomes.

## Introduction

Tuberculosis (TB) is a chronic infectious disease caused by *Mycobacterium tuberculosis*, primarily affecting the lungs (Pulmonary TB) and occasionally extending to other organs (Extra-Pulmonary TB) [1]. The threat of tuberculosis (TB) remains a persistent global issue, causing significant morbidity and mortality, particularly in low and middle-income countries [2].

In Ghana, the reported TB incidence rate was 133 per 100, 000 population in 2022 [3], a reduction from 136 per 100,000 population in 2021 [3,4], with the disease affecting every region, district, and community [5]. In 2020 the incidence and mortality rate of TB in Ghana was 143 and 49 per 100,000 population respectively [6]. The TB mortality rate decline from 49 in 2022–36 per 100, 000 population in 2021, and further decreased to 32 per 100,000 in 2022 [4,7]. Meanwhile, the increasing burden of TB highlights the need for effective disease management and treatment, including adherence to scheduled follow-up appointments. However, loss to follow-up (LTFU) of TB cases is a well-established obstacle to successful disease control and elimination efforts. LTFU may occur due to various factors, including death, limited accessibility to healthcare, stigma, drug side effects, prolonged therapy, inadequate support from treatment supporters, socio-economic factors, or environmental challenges [5]. Failure to complete TB treatment can lead to complications, drug resistance, and further transmission of the disease. LTFU can also result in decreased TB treatment success rates, increased risk of clinical deterioration, and treatment failure [8]. LTFU is a pivotal indicator for public health officers and epidemiologists as it can significantly influence both the incidence and prevalence of TB cases at local, national, and global levels.

Some studies have explored various aspects of TB LTFU, including factors associated with LTFU in low-incidence regions [9], characteristics and determinants of LTFU among TB patients who smoke [10], and factors associated with LTFU before and after treatment initiation [11]. However, majority of studies on this subject [12], especially in Africa [13,14], have not utilized spatial analysis techniques. Existing studies on LTFU in Ghana have mainly focused on specific regions or healthcare facilities, limiting the generalizability of the findings [15,16]. Analyzing spatiotemporal patterns of tuberculosis (TB), with the appropriate spatial analysis techniques would enable policy makers to address LTFU hotspots areas in Ghana. Notably, this depends on the research aim, the nature of the data, and available resources. For instance, Spatial Autoregressive (SAR) incorporates the influence of neighboring regions, which is crucial for infectious diseases like TB, but shortfalls is that it requires significant assumption about spatial clusters and intensive computational with large data [17]. The Bayesians Spatiotemporal handles uncertainty and small samples adequately while improving estimates of sparse data, however the results are sensitive to prior specification [18–20]. Spatial intensity patterns of TB cases can effectively visualize using Kernel density but this followed a non-parametric distribution which does not account for temporal dynamics, and the results may also be affected due to bandwidth selection [21,22].While Geographically Weighted Regression (GWR) and Space-Time Scan can identify spatial variation in relationships and detect or test clusters in space and time, they are also faced multicollinearity issues, fixed cluster shape and size or may be sensitive to input parameters [23–26]. This study aims to geospatially analyze LTFU of TB cases at the district level in Ghana using spatial-temporal analysis techniques. However, to leveraged the strengths of each while mitigating individual limitations, this study relied on the Global Moran’s, LISA and Getis-Ord G* to provide robust findings on TB’s spatiotemporal dynamics. Though, these methods does not provide causal inferences as well as not applicable in heterogenous clusters due to stationarity, they are easy to compute and visualize spatial autocorrelation [27,28]. Besides, the nature of this data requires direct computation as there is are individual factors. Therefore, identifying hotspots and spatial patterns of TB LTFU, this research will provide valuable insights the development of more effective, targeted strategies for the National Tuberculosis Programs to reduce LTFU and improve overall TB treatment outcomes in Ghana.

## Methods

### Study design

This study employed a cross-sectional design to assess the distribution of tuberculosis patients lost to follow-up care among districts in Ghana.

### Study area

**Fig 1:**
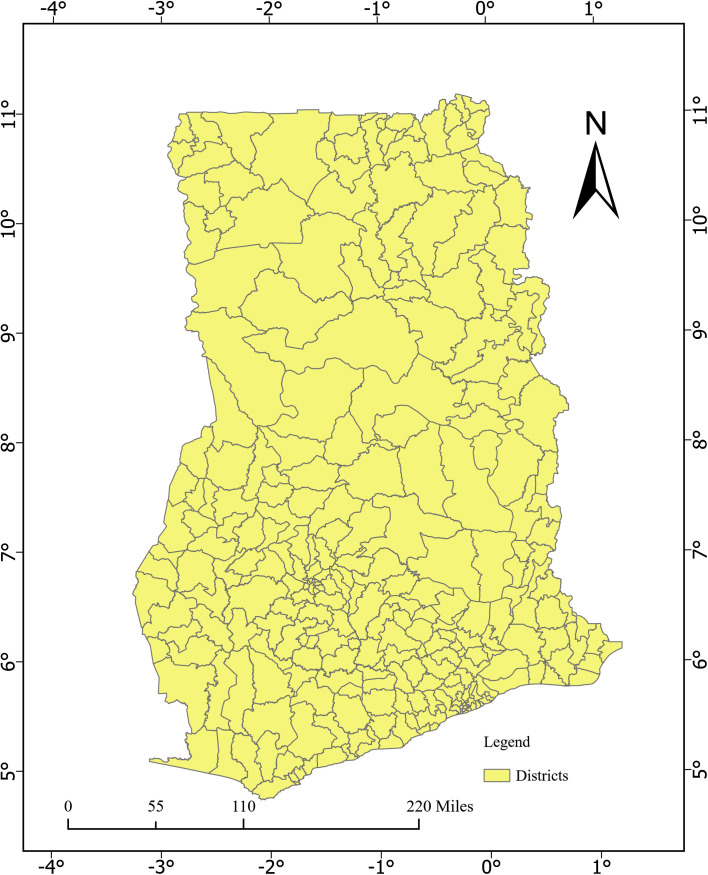
Map of Ghana with the 261 administrative districts.

It’s covered all 261 districts across the 16 administrative regions of Ghana as shown in Fig 1. Ghana is situated in West Africa and is bordered by Togo to the east, Cote d’Ivoire to the west, to the north and south is Burkina Faso, and the Gulf of Guinea accordingly. Reports of the 2021 Population and Housing Census (PHC) shows that the country’s population is 30,832,019 million [29]. Representing 50.7% of females and 49.3% of males. The majority 56.7% of the country’s population is in the urban areas with 43.3% living in the rural areas. The total land area of Ghana is 238,533 sq km with latitudes ranging from 4.74° N to 11.17° N and longitudes between −3.26° W and 1.19° E. The temperature is hot and humid in the southwest but hot and dry in the north whereas the climate in the southeast show dry and warm seasons. The climate variability including social, political and economic factors encourages internal mobility in the country. The Ghanaian health system is decentralized, consisting of national, regional, district, sub-district, and community levels. The Ministry of Health formulate policies, while implementation is carried out by the Ghana Health Service, the Christian Health Association of Ghana, and other private institutions.

### Ethical consideration and participation consent

We sought data approval from the Ashanti Regional Health Directorate. The study was also approved by the Committee on Human Research, Publication and Ethics, KNUST Ref. CHRPE/AP/1000/24. The study uses aggregated data of TB lost to follow up cases, so participant’s consent was not needed. That is, the dataset did not contain any pertinent participant sociodemographic information.

### Data source and management

The study utilized secondary data extracted from the District Health Information Management System (DHIMS2) database in Ghana. DHIMS2 is an electronic tool deployed by the Ministry of Health to capture aggregated data on communicable and non-communicable diseases, as well as other public health-related issues. It consolidates data from various health sectors into a single location. Data on TB LTFU cases from 1^st^ January, 2019–31^st^ December, 2023 were extracted from DHIMS2.

For this study, a TB LTFU case was defined as a TB patient who had been diagnosed and put on treatment for at least four weeks and then discontinued treatment. Cases due to death were excluded from the analysis.

The extracted data were processed using Microsoft Excel to ensure compatibility with ArcGIS Pro version 3.1.0 under Copyright © 2023 Esri Inc. Districts in the dataset were matched with those in the ArcGIS Pro shapefile, and a Field Identification number (FID) was generated for each district. Blank observations were replaced with zeros. The processed data were then merged with the shapefile using the FID.

### Data analysis

The proportions of lost to follow-up cases were used to analyze the data. This was determined by dividing lost to follow-up cases at every district in each year by the total number of TB cases in each year multiplied by 100 percent. However, the data was transformed using a log function for each year. Map visualization was carried out in ArcGIS Pro to visually represent the locations of lost to follow up on TB cases and analyze their spatial features. The digitalized maps were converted into images and saved in Portable Document Format. To gain insights into the spatiotemporal patterns of LTFU, we use spatial statistical mapping techniques, because it involves several procedures for examining and modeling spatial data. In this process we used a map scale of 1:7000000. LTFU can be performed using spatial analysis because a spatial database is a complete database that contains all the required information. This database is regularly updated through field verification and generates data layers from diverse available sources. The study uses spatial statistical analysis approaches such as; the Global Moran’s I, and Getis-Ord Gi* to perform spatial analysis, create maps, and generate graphs. The findings of this study are relevant for decision-makers as it help identify hotspot locations and guide resource allocation for improving healthcare services.

#### Spatial autocorrelation.

Spatial autocorrelation analysis is a powerful spatial statistical approach that shows district locations of spatial patterns. It usually measures the association between an element attribute and attribute point in the nearest spatial point. Lost to follow-up were analyzed for their spatial autocorrelation using global Moran’s I. The Moran’s I statistic is robust in detecting the presence of a spatial pattern amongst a variable [30]. The presence of a spatial pattern in the distribution of TB cases was detected through Moran’s I in studies by [1,31]. It is likely to determine the concentration levels and the type of spatial pattern—clustering, dispersion, or random—by calculating Moran’s I. The value of Moran’s I is between −1 and 1. There exist positive and negative clustering if the Moran’s I value is positive and negative respectively, and no clustering if the value is zero. The null hypothesis presumed that there is randomness. At this point, we used Euclidean method with Moran’s I distance threshold of 41.66 Miles. The Moran I Index is mathematically stated as;


$$I(h)=\;\frac{1}{s^{2}}\frac{\mathrm{\backslash sumi}\mathrm{\backslash sumj}(y_{i}-\overset{―}{y})(y_{j}-\overset{―}{y})}{\mathrm{\backslash sumi}{\mathrm{\backslash sumjw}}_{ij}}$$ (1)

Where, $y_{i}$ is the data value at location *i*, *h* is the distance between locations *i* and *j*, $w_{ij}$ takes 1 if the pair (*i*, *j*) pertains to distance class *h* (the one for which the coefficient is computed), otherwise 0. Where, W is the sum of $w_{ij}$ [32], and y is the dependent variable, $\overset{―}{y}$ is the mean, $s^{2}$ is the variance, and w is the binary connectivity matrix.

#### Optimized cluster and outlier analysis.

To determine the optimized cluster and outliers the study adopted the Local Indicators of Spatial Association (LISA). The LISA, which measure the degree of spatial association at each distinct place, offer a more thorough examination of spatial autocorrelation than global Moran’s I, which assesses spatial autocorrelation as a whole [27]. LISA is special tool to spot and examine spatial samples at a neighborhood level in a dataset. Under this analysis five possible outcomes are expected thus; high-high, low-low, low-high, high-low, and not significant. The high-high indicates a high clustering and low-low show diffusion of cases in a specific district whereas low-high and high-low indicates probable geographical outliers. The mathematical formula for LISA can be stated as:


$$I_{i}=\;\frac{n\;(x_{i}-\overset{―}{x})\sum_{j=1}^{n}w_{ij}(x_{j}-\overset{―}{x})}{(n-1)S^{2}}$$ (2)

From the equation, n represents the total number of lost to follow up of districts. The variables $x_{i}$ and $x_{j}$ denotes the observations at locations i and j, respectively. $\overset{―}{x}$ is the mean of the observations. The element Wij belongs to the spatial weights matrix w and represents the spatial weight between locations i and j. Those with a significant proportion of TB lost to follow up cases marked as hotspots, and locations with a limited incidence of lost to follow up cases shows a cold spot. Areas with combination of elevated and minimal lost to follow up incidence in nearby districts were the studied as spatial outliers.

#### Hotspot analysis.

The Getis-Ord Gi* statistics are a group of statistical measures that possess several desirable properties for quantifying spatial dependence in a variable that is distributed across space. We adopted Getis-Ord G* to analysis hotspot areas. We used Gi-Bin values ranging from −3 to +3, the confidence levels 90%, 95% and 99% corresponds with z-scores values ±1.65, ± 1.96 and ±2.58 accordingly. In this case, a z ≥ 1.65 was considered as hotspot while z ≤ 1.65 was recorded coldspot. This study relies on the highest confidence level (99%) to identify significant hotspot districts of lost to follow up TB cases. The mathematical expression of Gi* statistic is shown below:


$$G_{i}^{*}=\frac{\sum_{j=1}w_{ij}x_{j}-\overset{―}{x}\sum_{j=1}w_{ij}}{S\sqrt{\frac{[n\sum_{j=1}w_{ij}^{2}-(\sum_{j=1}w_{ij}{)}^{2}]}{n-1}}}$$ (3)

where; $\overset{―}{X}=\frac{\sum_{j=1}^{n}x_{j}}{n}$, $S\;=\;\sqrt{\frac{\sum_{j=1}^{n}x_{j^{2}}}{n}}\;–\;\overset{―}{X}$

Here, “n” represents the total count of lost to follow-up, “S” denotes the standard deviation of the ratio of lost to follow-up cases, and “$w_{ij}$ (d)” for a function that returns 1 if the distance between location *j* and *i* is less than given distance “d,” and 0 in the other case. Again, “Sij” and “Wi*” are involved. The degree of influence that location *i* has within the specified distance “d” is represented by the magnitude of “Gi*”, which suggests that the location *i* (a district) is a hotspot area. A higher value of “Gi*” suggests a stronger influence and further establishes the significance of location *i* as a hotspot.

## Results

Over the five-year study period from 2019 to 2023, a total of 75,604 TB cases were recorded in Ghana, of which 2,887 were lost to follow-up (LTFU) cases.

### Temporal trends in TB LTFU

**Fig 2:**
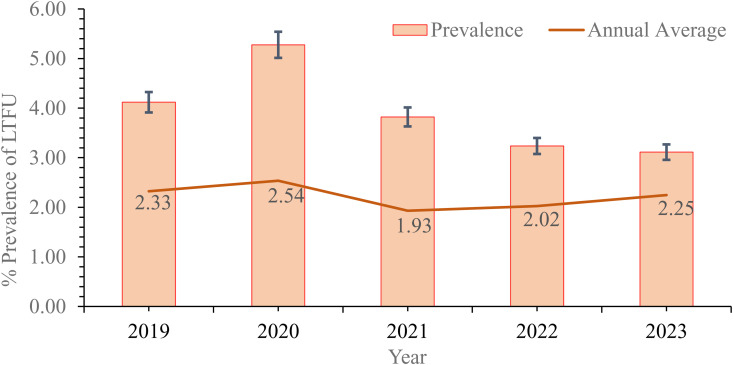
Prevalence of TB lost to follow-up cases in Ghana from 2019-2023.

The prevalence of TB LTFU cases in Ghana showed a fluctuating trend over the study period. As illustrated in Fig 2, the prevalence rate initially increased from 4.12% in 2019 to 5.28% in 2020. However, from 2020 onwards, a consistent decreasing trend was observed, with the prevalence dropping to 3.82% in 2021, 3.24% in 2022, and finally 3.11% in 2023. These figures, bounded by a 5% error margin, suggest an overall improvement in TB follow-up rates over the latter part of the study period. The annual average of TB LTFU was peaked in 2020 at 2.54, declined to 1.93 in 2021 and then gradually increased through 2023 as shown in Fig 2.

### Spatial distribution of TB LTFU

**Fig 3:**
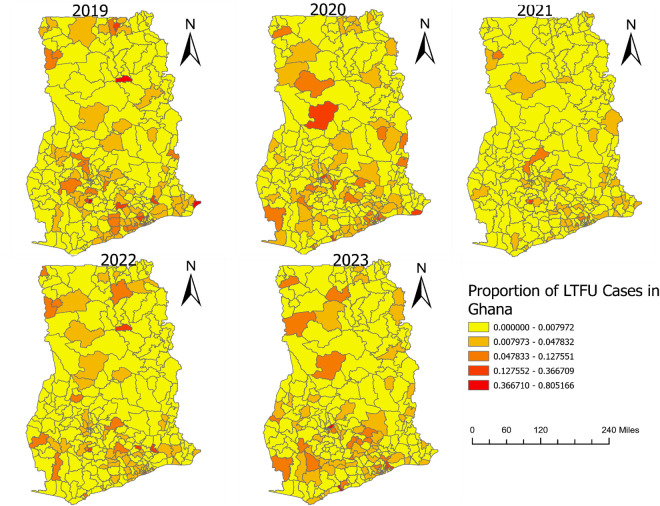
Proportional distribution of TB LTFU among districts from 2019-2023.

The proportional distribution of TB LTFU among districts, as shown in Fig 3, exhibited varying patterns across the years. In 2019, high densities of LTFU were observed in districts across Upper East, Volta, Central, Northern, Eastern, and Greater Accra regions. The year 2020 saw high densities in the Northern, Ashanti, Central, Eastern, and Greater Accra regions. Interestingly, 2021 showed a generally low density of TB LTFU cases across the country, with only a few districts in Upper West and Ashanti regions showing high densities. The pattern changed again in 2022, with high clustered cases identified in districts within Upper West, North East, Northern, Ashanti, Eastern, Central, and Greater Accra regions. In 2023, high density of LTFU cases were recorded in districts in Greater Accra, Central, Upper West, North East, Northern, and Ashanti regions.

### Spatial autocorrelation of TB LTFU

The spatial autocorrelation analysis, presented in Table 1, revealed significant changes in the spatial distribution of LTFU cases over the years. From 2019 to 2021, there was significant spatial clustering of LTFU cases, with Moran’s I values ranging from 0.0578 to 0.1078. Thus, the spatial autocorrelation pattern in 2019 was [Moran’s I = 0.10, z = 3.87; p < 0.0001], in 2020 [Moran’s I = 0.06, z = 2.23; p = 0.026] and 2021[Moran’s I = 0.11, z = 4.04; p < 0.0001]. However, in 2022 and 2023, the pattern shifted towards a more random distribution of LTFU cases across districts, with Moran’s I values of 0.0038 (p = 0.166) and 0.0361 (p = 0.149) respectively. This change suggests a transition from localized concentrations of LTFU cases to a more dispersed pattern in the latter years of the study.

**Table 1:** Spatial autocorrelation of TB LTFU in Ghana from 2019-2023.

Year	Moran’s Index	Z-score	P-value	Pattern
2019	0.1032	3.8694	0.001	Clustered
2020	0.0578	2.2313	0.026	Clustered
2021	0.1078	4.0387	0.001	Clustered
2022	0.0038	1.3856	0.166	Random
2023	0.0361	1.4448	0.149	Random

Source: Author’s Work (2024)

### Optimum clusters of TB LTFU

**Fig 4:**
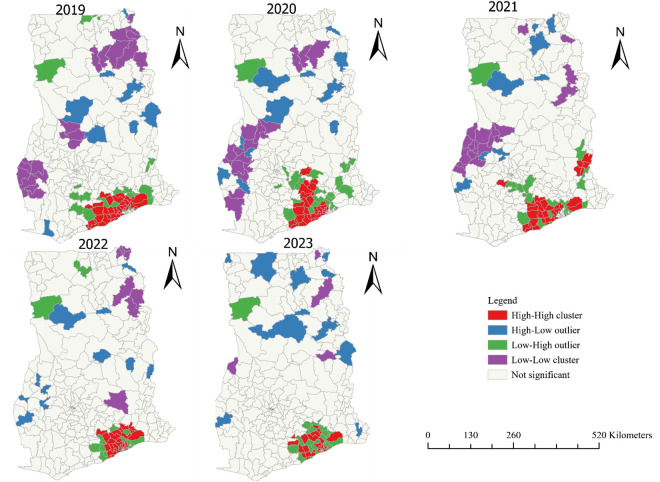
Optimized analysis of TB LTFU among districts in Ghana from 2019-2023.

After determine spatial autocorrelation, it was deemed appropriate identify districts with high cases. Fig 4 show that, in 2019 most high-high clusters were reported from 34 districts within Greater Accra, Eastern and Central region. For the year 2020, high-high clusters were also located in 28 districts in these same regions. Meanwhile, in 2021 LFTU cases were extremely clusters in 30 districts across Greater Accra, Central, Eastern, Volta and Ashanti regions. The Fig 4 further show that, highly clustered LFTU cases were in 32 districts within three regions (Greater Accra, Central and Eastern) and 33 districts in Greater Accra, Central and Eastern in 2022 and 2023 respectively. Notwithstanding, most districts in Western, Bono, Savanna, Northern and Ashanti regions found with very low TB LTFU cases in 2019, 2020, and 2021.

### Hotspots analysis of TB LTFU

**Fig 5:**
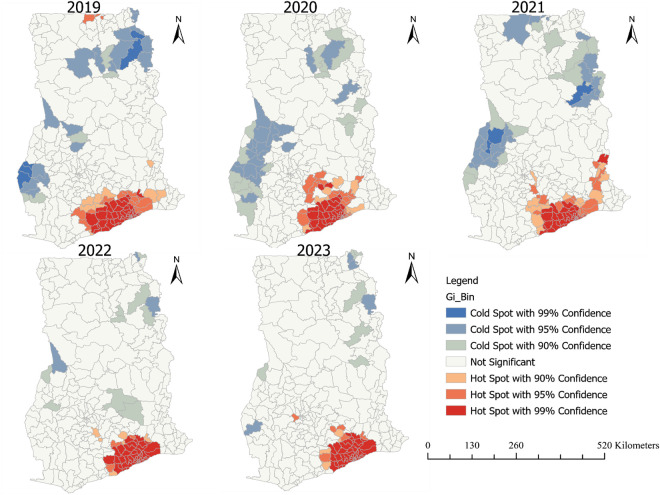
Hotspot analysis of TB LTFU among districts in Ghana from 2019-2023.

The hotspot analysis, illustrated in Fig 5, provided further insights into the spatial concentration of LTFU cases. Focusing on districts with hotspots at a 99% confidence level, the analysis revealed that the number and distribution of hotspot districts varied over the years. In 2019, 48 hotspot districts were identified across three regions: Greater Accra (23), Central (15), and Eastern (10). S1 File Hotspot analysis of TB LTFU from 2019 to 2023. The number of hotspot districts decreased slightly to 38 in both 2020 and 2021, but with varying regional distributions (see S1 Table in S1 File). In 2022, the number of hotspot districts increased to 49, with a notable concentration in Greater Accra (27), Eastern (13), and Central (9) regions (see S2 Table in S1 File). The pattern remained similar in 2023, with 48 hotspot districts identified in the same regions (see S2 Table in S1 File). Throughout the study period, certain districts consistently emerged as hotspots. These included districts in Greater Accra such as Ga East Municipal, Ayawaso West Municipal, and Ga West Municipal; in the Central region, districts like Gomoa East, Gomoa Central, and Asikuma-Odoben-Brakwa; and in the Eastern region, districts such as Nsawam-Adoagyiri Municipal and Akwapim South. The persistence of these hotspots suggests that these areas may require targeted interventions to address the ongoing challenge of TB LTFU. Meanwhile, districts exhibiting hotspots may liaise with districts with coldspot of TB LFTU such as Bimbila and Gushegu in Northern region, Dormaa West, Tian and Berekum East in the Bono region, Yuyoo in North East region as well as Bia West, and Bia East in the Western North region.

## Discussion

The findings of this study provide valuable insights into the spatiotemporal patterns of tuberculosis (TB) lost to follow-up (LTFU) cases in Ghana from 2019 to 2023. The overall trend in TB LTFU prevalence shows an initial increase followed by a consistent decline, which differs from previous reports. [13] reported an increasing trend of TB LTFU from 2012 to 2021 in Ghana, contrary to our findings. The spike in LTFU cases observed in 2020 could be attributed to the COVID-19 pandemic, which likely disrupted healthcare services and patient follow-up. The subsequent decline in LTFU rates from 2021 to 2023 suggests an improvement in TB management strategies, possibly due to the intensification of efforts by treatment supporters or enhanced interventions by the National Tuberculosis Programs [33]. The proportional distribution and hotspot analysis consistently identified high densities of LTFU cases in densely populated districts in regions such as Greater Accra, Central, and Eastern regions. This pattern mirrors findings from a study by [34], who reported that most TB patients in Bandar Lampung, Indonesia, lived in congested housing and overcrowded neighborhoods, increasing the risk of TB transmission. In Ghana, these regions are characterized by high population density, with some districts associated with poor housing conditions and environmental hygiene, factors known to influence TB transmission and treatment adherence [29]. The fluctuating patterns of LTFU across different regions and years underscore the complex nature of TB control. Factors such as rural-urban migration, as noted by [5], can complicate patient follow-up [5]. The six-month duration of TB treatment, requiring frequent hospital visits, poses challenges for non-residents or migrant workers in urban centers. This aligns with findings by [8,11], who identified employment status as a factor associated with LTFU.

The spatial autocorrelation analysis revealed significant clustering of LTFU cases from 2019 to 2021, followed by a shift towards a more random distribution in 2022 and 2023. This spatial dependency, particularly in the southern regions of Ghana, aligns with findings from other studies. [31]suggested that such clustering might be facilitated by convenient transportation or migration. The transition to a more random distribution in later years could indicate improvements in TB control efforts across the country, reducing localized concentrations of LTFU cases. The persistence of certain districts as LTFU hotspots throughout the study period is particularly concerning. This consistent pattern suggests that these areas face ongoing challenges in TB patient retention and follow-up. Similar to findings by [35] in southern Ethiopia, where LTFU was more frequently observed among patients from rural areas, the results of this study highlight the need for targeted interventions in specific districts. The concentration of hotspots in urban and peri-urban areas of Ghana also aligns with observations by [8,36], who associated LTFU with residence type. The identification of consistent hotspot areas provides crucial information for policymakers and health officials. As suggested by [12], the features of high-risk areas for TB LTFU and factors affecting its hotspot locations are often related to poor socioeconomic conditions, high population density, and the spread of risk to neighboring areas. It would therefore be critical for stakeholders, policy-makers and/or agencies to improve TB care and control. Thus, an immediate action for them is to enhanced understanding of TB LTFU dynamics. To begin, we advised stakeholders to provide clinicians and programs implementers with adequate information on the motives and risk factors for TB patients discontinuing treatment. For instance, [11] realized that comprehensive information and address concerns to improve patients’ awareness and attitudes towards TB care was crucial, after observing that adverse reactions in TB patients increase LTFU risk. This study also suggest that policy makers offer evidence on the gaps in the health system, such as accessibility, quality of care, and socio-economic barriers, and enabling informed policy adjustments. In addition, Ghana Health Service including agencies should support donors and international organization with insights on how investment in TB care programs is impacting patients’ retentions. Aside, it is recommended that stakeholders to empower health advocate by pushing for patient-centered models and address social determinants of health in TB programs. This study also beliefs that digital adherence technology can improve LTFU therefore policy makers and agencies such as the National Tuberculosis Program Agency should provide support. This would enable global TB initiatives align their priorities with local challenges, fostering a more cohesive approach to TB care in Ghana. Nonetheless, this study revealed that interventions aimed at reducing LTFU rates can develop standardized approaches and indicators for LTFU and establishing benchmark for program performance and patient retention. The findings of the study also contribute to economic and social implications. Thus, policy makers can use this evidence to justify investment in social protection scheme for TB patients in the affected districts. Meanwhile, stakeholders may rely on this fact to advocate for funding in preventive strategies as TB LTFU can occur due non-completion of treatment, treatment cost or drug resistance. Finally, our study contributes to Sustainable Development Goals in that improving treatment outcomes can lead to good health and well-being, and reduced poverty by minimizing the economic burden on patients and families.

## Conclusions

This study provides a spatiotemporal analysis of Tuberculosis (TB) Lost to Follow-Up (LTFU) cases in Ghana from 2019 to 2023, revealing complex patterns across the country. We observed an initial increase followed by a consistent decline in LTFU prevalence rates, with spatial analysis identifying significant clustering in earlier years transitioning to a more random distribution later. Persistent hotspots were identified primarily in densely populated regions, emphasizing the need for targeted interventions in these areas. This study underscores the importance of considering spatial and temporal factors in TB control strategies and provide valuable information for policymakers to allocate resources more effectively.

### Limitations

Even though this study appears as the first in research, constraints are not different from other studies. That is, aggregated data from DHIMS2 was used for spatial mapping without considering probable indicators that are associated with TB LTFU. Therefore, future studies should consider factors that are associated with TB LTFU.

## Supporting information

S1 File Hotspot analysis of TB LTFU from 2019 to 2023. (Table 1) Hotspot Districts of TB LTFU from 2019 to 2021. (Table 2) Hotspot Districts of TB LTFU from 2022 to 2023.
(DOCX)

## References

[1] Yu Y, Wu B, Wu C, Wang Q, Hu D, Chen W. Spatial-temporal analysis of tuberculosis in Chongqing, China 2011-2018. BMC Infect Dis. 2020;20(1):531. doi: 10.1186/s12879-020-05249-3 32698763 PMC7374877

[2] WHO. Global tuberculosis report 2023. Geneva: World Health Organization. 2023. https://iris.who.int/bitstream/handle/10665/373828/9789240083851-eng.pdf?

[3] The Global Economy. Ghana tuberculosis-data, chart. Accessed 2024 December 3. https://www.theglobaleconomy.com/Ghana/Tuberculosis/.

[4] WHO. Country disease outlook Ghana. WHO African region. 2023. https://www.google.com/search?q=prevalenceofcommunicablediseasesinMalawi&sca_esv=590391945&rlz=1C1ONGR_enMW1082MW1083&sxsrf=AM9HkKkXagPDZLEiLcBGhAs6SkpoJcqZw%3A1702435482663&ei=mhp5ZfaTKIyNkdUP5pu1wAk&ved=0ahUKEwj2yMmxsouDAxWMRqQEHeZNDZgQ4dUDCBA&uac

[5] Bio RB, Akweongo P, Adomah-Afari A, Koduah A. Determinants of tuberculosis treatment support costs to the treatment supporters in rural Ghana. AIMS Public Health. 2023;10(1):78–93. doi: 10.3934/publichealth.2023007 37063356 PMC10091134

[6] Tuberculosis: the noguchi memorial institute for medical research. The Noguchi memorial institute. Accessed 2024 December 3. https://noguchi.ug.edu.gh/main-research/tuberculosis/

[7] Trading Economics. Ghana - incidence of tuberculosis (per 100,000 People): 2024 data 2025 forecast 1990-2022 historical [Internet]. Accessed 2024 December 3. https://tradingeconomics.com/ghana/incidence-of-tuberculosis-per-100-000-people-wb-data.html

[8] Rahayu SR, Susilastuti MS, Saefurrohim MZ, Azam M, Indrawati F, Supriyono M, et al. Lost to follow-up among tuberculosis patients during the public-private mix era in rural area of Indonesia. Ethiop J Health Sci. 2023;33(1):115–22. doi: 10.4314/ejhs.v33i1.15 36890941 PMC9987293

[9] Tetart M, Meybeck A, Assaf A, Valette M, Choisy P, Blondiaux N, et al. Factors of loss to follow-up during tuberculosis treatment in a low-incidence region. Med Mal Infect. 2020;50(1):28–35. doi: 10.1016/j.medmal.2019.02.007 30890281

[10] Sharani ZZ, Ismail N, Yasin SM, Zakaria Y, Razali A, Demong NAR, et al. Characteristics and determinants of loss to follow-up among tuberculosis (TB) patients who smoke in an industrial state of Malaysia: a registry-based study of the years 2013-2017. BMC Public Health. 2022;22(1):638. doi: 10.1186/s12889-022-13020-3 35365112 PMC8976383

[11] Jiang Y, Chen J, Ying M, Liu L, Li M, Lu S, et al. Factors associated with loss to follow-up before and after treatment initiation among patients with tuberculosis: a 5-year observation in China. Front Med (Lausanne). 2023;10:1136094. doi: 10.3389/fmed.2023.1136094 37181365 PMC10167013

[12] Teibo TKA, Andrade RL de P, Rosa RJ, Tavares RBV, Berra TZ, Arcêncio RA. Geo-spatial high-risk clusters of tuberculosis in the global general population: a systematic review. BMC Public Health. 2023;23(1):1586. doi: 10.1186/s12889-023-16493-y 37598144 PMC10439548

[13] Ababu DG, Gobena WE, Getahun AM. Prevalence of tuberculosis and the determinants of lose to follow-up the treatment for tuberculosis patients in case of Buno Bedele and Ilu Ababor Zones, Oromia, Ethiopia. Infect Drug Resist. 2022;15:5321–9. doi: 10.2147/IDR.S373230 36106054 PMC9467688

[14] Birhane M, Mekonnen S, Dingeta T, Teklemariam Z. Loss to follow-up tuberculosis treatment and associated factors among adults attending at public health facilities in Warder District, Somali Regional State, Eastern Ethiopia. Front Public Health. 2023;11:1151077. doi: 10.3389/fpubh.2023.1151077 37234759 PMC10208408

[15] Osei E, Oppong S, Adanfo D, Doepe BA, Owusu A, Kupour AG, et al. Reflecting on tuberculosis case notification and treatment outcomes in the Volta region of Ghana: a retrospective pool analysis of a multicentre cohort from 2013 to 2017. Glob Health Res Policy. 2019;4:37. doi: 10.1186/s41256-019-0128-9 31890895 PMC6916450

[16] Puplampu P, Kyeremateng I, Asafu-Adjaye O, Asare AA, Agyabeng K, Sarkodee R, et al. Evaluation of treatment outcomes among adult patients diagnosed with tuberculosis in Ghana: a 10 year retrospective review. IJID Reg. 2023;10:9–14. doi: 10.1016/j.ijregi.2023.11.004 38045863 PMC10687693

[17] Anselin L. Spatial econometrics: metodhs and models. Kluwer Academic Publishers; 1988. p. 284.

[18] Banerjee S, Carlin BP, Gelfand AE. Hierarchical modeling and analysis for spatial data. Second ed. Chapman and Hall/CRC. 2015.

[19] Lawson AB. Bayesian disease mapping: hierarchical modeling in spatial epidemiology. Interdisciplinary statistics series. Second ed. Charleston, South Carolina, USA: Chapman & Hall/CRC; 2013.

[20] Lawson AB. Bayesian disease mapping: hierarchical modeling in spatial epidemiology. Interdisciplinary statistics series. Third ed. Chapman & Hall/CRC; 2018.

[21] Silverman B. Density estimation for statistics and data analysis. London: Chapman and Hall; 1986.

[22] Lin Y-P, Chu H-J, Wu C-F, Chang T-K, Chen C-Y. Hotspot analysis of spatial environmental pollutants using kernel density estimation and geostatistical techniques. Int J Environ Res Public Health. 2011;8(1):75–88. doi: 10.3390/ijerph8010075 21318015 PMC3037061

[23] Fotheringham AS, Brunsdon C, Charlton M. Geographically weighted regression: the analysis of spatially varying relationships. First ed. Chichester, England: Wiley; 2002.

[24] Lu B, Charlton M, Harris P, Fotheringham AS. Geographically weighted regression with a non-Euclidean distance metric: a case study using hedonic house price data. Inter J Geograph Inform Sci. 2014;28(4):660–81. doi: 10.1080/13658816.2013.865739

[25] Kulldorff M, Athas WF, Feurer EJ, Miller BA, Key CR. Evaluating cluster alarms: a space-time scan statistic and brain cancer in Los Alamos, New Mexico. Am J Public Health. 1998;88(9):1377–80. doi: 10.2105/ajph.88.9.1377 9736881 PMC1509064

[26] Kulldorff M. A spatial scan statistic. Communications in statistics - theory and methods. 1997;26(6):1481–96. doi: 10.1080/03610929708831995 PMC3867306 24363487

[27] Anselin L. Local indicators of spatial association—LISA. Geographical Analysis. 1995;27(2):93–115. doi: 10.1111/j.1538-4632.1995.tb00338.x

[28] Anselin L, Syabri I, Kho Y. GeoDa: an introduction to spatial data analysis. Geographical Analysis. 2005;38(1):5–22. doi: 10.1111/j.0016-7363.2005.00671.x

[29] Ghana Statistical Service. Ghana 2021 population and housing census: general report volume 3A-population of regions and districts. 2021. https://statsghana.gov.gh/gssmain/fileUpload/pressrelease/2021%20PHC%20General%20Report%20Vol%203A_Population%20of%20Regions%20and%20Districts_181121.pdf

[30] Dubé J, Legros D. Spatial autocorrelation. In: Spatial econometrics using microdata. Wiley; 2014. 59–91. doi: 10.1002/9781119008651.ch3

[31] Xia L, Zhu S, Chen C, Rao Z-Y, Xia Y, Wang D-X, et al. Spatio-temporal analysis of socio-economic characteristics for pulmonary tuberculosis in Sichuan province of China, 2006-2015. BMC Infect Dis. 2020;20(1):433. doi: 10.1186/s12879-020-05150-z 32571231 PMC7310234

[32] Fischer MM, Getis A. Handbook of applied spatial analysis. Springer Berlin Heidelberg; 2010. doi: 10.1007/978-3-642-03647-7

[33] NTP-Ghana. Ghana National TB programme report. 2023.

[34] Wardani DWSR, Wahono EP. Predominant determinants of delayed tuberculosis sputum conversion in Indonesia. Indian J Community Med. 2019;44(1):53–7. doi: 10.4103/ijcm.IJCM_319_18 30983715 PMC6437790

[35] Akessa GM, Tadesse M, Abebe G. Survival analysis of loss to follow-up treatment among tuberculosis patients at Jimma university specialized hospital, Jimma, Southwest Ethiopia. International Journal of Statistical Mechanics. 2015;2015:1–7. doi: 10.1155/2015/923025

[36] Kim HW, Park S, Min J, Sun J, Shin AY, Ha JH, et al. Hidden loss to follow-up among tuberculosis patients managed by public-private mix institutions in South Korea. Sci Rep. 2022;12(1):12362. doi: 10.1038/s41598-022-16441-7 35859107 PMC9300674

