# Supplementary material for: Spatiotemporal hotspot analysis of tuberculosis lost to follow-up cases in Ghana: A district-level study from 2019–2023
Source: PLoS One. 2025 Jul 2;20(7):e0326444. doi: 10.1371/journal.pone.0326444 (PMC12221003; doi:10.1371/journal.pone.0326444)
Supplement: S1 File — (Table 1) Hotspot Districts of TB LTFU from 2019 to 2021. (Table 2) Hotspot Districts of TB LTFU from 2022 to 2023. (DOCX) [file pone.0326444.s001.docx]

**Supplementary Information**

**Hotspots Analysis of TB LTFU 2019 to 2023**

Table 1: Hotspot Districts of TB LTFU from 2019 to 2021

| Region | 2019 | | 2020 | | 2021 | |
| --- | --- | --- | --- | --- | --- | --- |
|  | #Hotspot | District | #Hotspot | District | #Hotspot | District |
| Central | 15 | Agona East, Asikuma-Odoben-Brakwa, Agona West Municipal, Gomoa West, Effutu Municipal, Gomoa East, Gomoa Central, Awutu Senya, Awutu Senya East Municipal, Mfantsiman Municipal, Ekumfi, Ajumako-Enyan-Esiam, Achiase, Assin South, Abura-Asebu-Kwamankese | 13 | Asikuma-Odoben-Brakwa, Mfantsiman Municipal, Ekumfi, Ajumako-Enyan-Esiam, Agona West Municipal, Achiase, Gomoa West, Agona East, Effutu Municipal, Gomoa East, Gomoa Central, Awutu Senya, Awutu Senya East Municipal | 13 | Asikuma-Odoben-Brakwa, Mfantsiman Municipal, Ekumfi, Ajumako-Enyan-Esiam, Agona West Municipal, Achiase, Gomoa West, Agona East, Effutu Municipal, Gomoa East, Gomoa Central, Awutu Senya, Awutu Senya East Municipal |
| Eastern | 10 | Lower West Akim Municipal, Asene-Manso-Akroso, Upper West Akim, New Juaben North Municipal, Nsawam-Adoagyiri Municipal, Akwapim South, Suhum Municipal, Ayensuano, New Juaben South Municipal, Lower Manya Krobo | 10 | Lower West Akim Municipal, Atiwa East, Asene-Manso-Akroso, Upper West Akim, New Juaben North Municipal, Nsawam-Adoagyiri Municipal, Akwapim South, Suhum Municipal, Ayensuano, New Juaben South Municipal | 5 | Asene-Manso-Akroso, Upper West Akim, Nsawam-Adoagyiri Municipal, Akwapim South, Ayensuano |
| Greater Accra | 23 | Ga East Municipal, Ayawaso West Municipal, Ga West Municipal, Ga Central Municipal, Ayawaso East Municipal, Ayawaso North Municipal, Ayawaso Central Municipal, Ablekuma West Municipal, Ablekuma Central Municipal, Ga North Municipal, Weija-Gbawe Municipal, Ablekuma North Municipal, Ga South Metro, Accra Metro, Okaikwei North Municipal, Krowor, Korle Klotey, Ledzokorkor, La-Dade Kotopon Municipal, Adentan Municipal, La-Nkantanang-Madina Municipal, Tema West Municipal, Tema metro | 15 | Ga East Municipal, Ayawaso West Municipal, Ga West Municipal, Ga Central Municipal, Ayawaso East Municipal, Ayawaso North Municipal, Ayawaso Central Municipal, Ablekuma West Municipal, Ablekuma Central Municipal, Ga North Municipal, Weija-Gbawe Municipal, Ablekuma North Municipal, Ga South Metro, Accra Metro, Okaikwei North Municipal | 19 | Ga East Municipal, Ayawaso West Municipal, Ga West Municipal, Ga Central Municipal, Ayawaso East Municipal, Ayawaso North Municipal, Ayawaso Central Municipal, Ablekuma West Municipal, Ablekuma Central Municipal, Ga North Municipal, Weija-Gbawe Municipal, Ablekuma North Municipal, Ga South Metro, Accra Metro, Okaikwei North Municipal, Krowor, Korle Klotey, Ledzokorkor, La-Dade Kotopon Municipal |
| Volta |  |  |  |  | 1 | Hohoe Municipal |

Table 2: Hotspot Districts of TB LTFU from 2022 to 2023

| Region | 2022 | | 2023 | |
| --- | --- | --- | --- | --- |
|  | #Hotspot | District | #Hotspot | District |
| Central | 9 | Agona East, Asikuma-Odoben-Brakwa, Agona West Municipal, Gomoa West, Effutu Municipal, Gomoa East, Gomoa Central, Awutu Senya, Awutu Senya East Municipal | 9 | Agona East, Asikuma-Odoben-Brakwa, Agona West Municipal, Gomoa West, Effutu Municipal, Gomoa East, Gomoa Central, Awutu Senya, Awutu Senya East Municipal |
| Eastern | 13 | Lower West Akim Municipal, Asene-Manso-Akroso, Upper West Akim, New Juaben North Municipal, Nsawam-Adoagyiri Municipal, Akwapim South, Suhum Municipal, Ayensuano, New Juaben South Municipal, Abuakwa South Municipal, Okere, Yilo Krobo Municipal, Akwapim North Municipal | 13 | Lower West Akim Municipal, Upper West Akim, New Juaben North Municipal, Nsawam-Adoagyiri Municipal, Akwapim South, Suhum Municipal, Ayensuano, New Juaben South Municipal, Okere, Yilo Krobo Municipal, Akwapim North Municipal, Asene-Manso-Akroso, Abuakwa North |
| Greater Accra | 27 | Ga East Municipal, Ayawaso West Municipal, Ga West Municipal, Ga Central Municipal, Ayawaso East Municipal, Ayawaso North Municipal, Ayawaso Central Municipal, Ablekuma West Municipal, Ablekuma Central Municipal, Ga North Municipal, Weija-Gbawe Municipal, Ablekuma North Municipal, Ga South Metro, Accra Metro, Okaikwei North Municipal, Krowor, KORLE KLOTEY, Ledzokorkor, La-Dade Kotopon Municipal, Adentan Municipal, Shai Osudoku, Ningo Prampram, La-Nkantanang-Madina Municipal,Tema West Municipal, Ashaiman Municipal, Kpone-Katamanso Municipal,Tema metro | 27 | Ga East Municipal, Ayawaso West Municipal, Ga West Municipal, Ga Central Municipal, Ayawaso East Municipal, Ayawaso North Municipal, Ayawaso Central Municipal, Ablekuma West Municipal, Ablekuma Central Municipal, Ga North Municipal, Weija-Gbawe Municipal, Ablekuma North Municipal, Ga South Metro, Accra Metro, Okaikwei North Municipal, Krowor, Korle Klotey, Ledzokorkor, La-Dade Kotopon Municipal, Adentan Municipal, Shai Osudoku, Ningo Prampram,La-Nkantanang-Madina Municipal,Tema West Municipal, Ashaiman Municipal, Kpone-Katamanso Municipal,Tema metro |
